# Earlier snowmelt may lead to late season declines in plant productivity and carbon sequestration in Arctic tundra ecosystems

**DOI:** 10.1038/s41598-022-07561-1

**Published:** 2022-03-21

**Authors:** Donatella Zona, Peter M. Lafleur, Koen Hufkens, Barbara Bailey, Beniamino Gioli, George Burba, Jordan P. Goodrich, Anna K. Liljedahl, Eugénie S. Euskirchen, Jennifer D. Watts, Mary Farina, John S. Kimball, Martin Heimann, Mathias Göckede, Martijn Pallandt, Torben R. Christensen, Mikhail Mastepanov, Efrén López-Blanco, Marcin Jackowicz-Korczynski, Albertus J. Dolman, Luca Belelli Marchesini, Roisin Commane, Steven C. Wofsy, Charles E. Miller, David A. Lipson, Josh Hashemi, Kyle A. Arndt, Lars Kutzbach, David Holl, Julia Boike, Christian Wille, Torsten Sachs, Aram Kalhori, Xia Song, Xiaofeng Xu, Elyn R. Humphreys, Charles D. Koven, Oliver Sonnentag, Gesa Meyer, Gabriel H. Gosselin, Philip Marsh, Walter C. Oechel

**Affiliations:** 1 Department Biology, San Diego State University, San Diego, CA 92182 USA; 2 School of Biosciences, University of Sheffield, Western Bank, Sheffield, S10 2TN UK; 3 School of the Environment, Trent University, Peterborough, ON K9L 0G2 Canada; 4 UMR 1391 ISPA, INRA, 71 Avenue Edouard Bourlaux, 33140 Villenave d’Ornon, France; 5 Department of Applied Ecology and Environmental Biology, Ghent University, 653, 9000 Ghent, Belgium; 6 Institute of BioEconomy, IBE, National Research Council (CNR), Via Giovanni Caproni 8, 50145 Firenze, Italy; 7 LI-COR Biosciences, 4421 Superior St., Lincoln, NE 68504 USA; 8 The Robert B. Daugherty Water for Food Global Institute, School of Natural Resources, University of Nebraska, Lincoln, NE 68583 USA; 9 Department of Earth Sciences, University of Waikato, Hillcrest, Hamilton, 3216 New Zealand; 10 Water and Environmental Research Center, University of Alaska Fairbanks, Fairbanks, AK 99775-7340 USA; 11 Woodwell Climate Research Center, Falmouth, MA 02540 USA; 12 Institute of Arctic Biology, University of Alaska Fairbanks, Fairbanks, AK 99775 USA; 13 W.A. Franke College of Forestry & Conservation, The University of Montana, Missoula, MT 59812 USA; 14 Max Planck Institute for Biogeochemistry, 07745 Jena, Germany; 15 Institute for Atmospheric and Earth System Research (INAR)/Physics, Faculty of Science, University of Helsinki, Gustaf Hällströmin katu 2b, 00560, P.O. Box 64, 00014 Helsinki, Finland; 16 Department of Ecoscience, Arctic Research Centre, Aarhus University, Frederiksborgvej 399, 4000 Roskilde, Denmark; 17 Oulanka Research Station, Oulu University, Kuusamo, Finland; 18 Department of Environment and Minerals, Greenland Institute of Natural Resources, 3900 Nuuk, Greenland; 19 Department of Physical Geography and Ecosystem Science, Lund University, 22362 Lund, Sweden; 20 The Netherlands Institute for Sea Research, Den Burg, PO Box 59, 1790 AB Texel, The Netherlands; 21 Department of Sustainable Agro-Ecosystems and Bioresources, Research and Innovation Centre, Fondazione Edmund Mach, Via E. Mach 1, 38010 San Michele all’Adige, TN Italy; 22 Agrarian‐Technological Institute, RUDN University, 117198 Moscow, Russia; 23 Department of Earth and Environmental Sciences, Lamont-Doherty Earth Observatory, Columbia University, Palisades, NY 10964 USA; 24 School of Engineering and Applied Sciences, Harvard University, 20 Oxford St., Cambridge, MA 02138 USA; 25 Jet Propulsion Laboratory, California Institute of Technology, 4800 Oak Grove Drive, Pasadena, CA 91109-8099 USA; 26 Earth Systems Research Center, Institute for the Study of Earth, Oceans, and Space, University of New Hampshire, 8 College Road, Durham, NH 03824 USA; 27 Institute of Soil Science, Center for Earth System Research and Sustainability (CEN), Universität Hamburg, 20146 Hamburg, Germany; 28 Permafrost Research, Alfred Wegener Institute, Helmholtz Centre for Polar and Marine Research, Potsdam, Germany; 29 Geography Department, Humboldt-Universität zu Berlin, 10099 Berlin, Germany; 30 GFZ German Research Centre for Geosciences, 14473 Potsdam, Germany; 31 Department of Geography and Environmental Studies, Carleton University, Ottawa, ON K1S 5B6 Canada; 32 Climate and Ecosystem Sciences Division, Lawrence Berkeley National Laboratory (LBNL), Berkeley, CA 94720 USA; 33 Département de Géographie, Université de Montréal, 1375 Avenue Thérèse-Lavoie-Roux, Montréal, QC H2V 0B3 Canada; 34 Department of Geography and Environmental Studies, Wilfrid Laurier University, 75 University Ave W., Waterloo, ON N2S 3C5 Canada; 35 Department of Geography, College of Life and Environmental Sciences, University of Exeter, Exeter, EX4 4RJ UK

**Keywords:** Ecology, Climate sciences, Environmental sciences

## Abstract

Arctic warming is affecting snow cover and soil hydrology, with consequences for carbon sequestration in tundra ecosystems. The scarcity of observations in the Arctic has limited our understanding of the impact of covarying environmental drivers on the carbon balance of tundra ecosystems. In this study, we address some of these uncertainties through a novel record of 119 site-years of summer data from eddy covariance towers representing dominant tundra vegetation types located on continuous permafrost in the Arctic. Here we found that earlier snowmelt was associated with more tundra net CO_2_ sequestration and higher gross primary productivity (GPP) only in June and July, but with lower net carbon sequestration and lower GPP in August. Although higher evapotranspiration (ET) can result in soil drying with the progression of the summer, we did not find significantly lower soil moisture with earlier snowmelt, nor evidence that water stress affected GPP in the late growing season. Our results suggest that the expected increased CO_2_ sequestration arising from Arctic warming and the associated increase in growing season length may not materialize if tundra ecosystems are not able to continue sequestering CO_2_ later in the season.

## Introduction

Climate change is affecting arctic ecosystems through temperature increase^1^, hydrological changes^2^, earlier snowmelt^3,4^, and the associated increase in growing season length^5^. Annual arctic air temperature has been increasing at more than double the magnitude of the global mean air temperature increase^1^, and terrestrial snow cover in June has decreased by 15.2% per decade from 1981 to 2019^4^. Warming is the main driver of the earlier start of the growing season, and the greening of the Arctic^6–8^. Arctic greening is associated with enhanced vegetation height, biomass, cover, and abundance^9^. However, the complexity of arctic systems reveals an intricate patchwork of landscape greening and browning^8,10,11^, with browning linked to a variety of stresses to vegetation^8^ including water stress^12,13^. The interconnected changes in temperature, soil moisture, snowmelt timing, etc. can have important effects on the carbon sequestered by arctic ecosystems^14^. The reservoir of carbon in arctic soil and vegetation depends on the interaction of two main processes: (1) changes in net CO_2_ uptake by vegetation; and (2) increased net loss of CO_2_ (from vegetation and soil) to the atmosphere via respiration. Therefore, defining the response of both plant productivity and ecosystem respiration to environmental changes is needed to predict the sensitivity of the net CO_2_ fluxes of arctic systems to climate change.

An earlier snowmelt, and a longer growing season, do not necessarily translate into more carbon sequestered by high latitude ecosystems^5^. There is a large disagreement on the response of plant productivity and the net CO_2_ uptake to early snowmelt in tundra ecosystems^15–19^. A warmer and longer growing season might not result in more net CO_2_ uptake if CO_2_ loss from respiration increases^16^, particularly later in the season, and surpasses the CO_2_ sequestered by enhanced plant productivity in northern ecosystems^16,20^. Moreover, snowmelt timing and the growing season length greatly affect hydrologic conditions of arctic soils^21^, as well as plant productivity^22^. Longer non-frozen periods earlier in the year^23^ and earlier vegetation greening can increase evapotranspiration (ET), resulting in lower summer soil moisture^24–26^. The complexity in the hydrology of tundra systems arises from the tight link between the water drainage and the presence and depth of permafrost. The presence of permafrost reduces vertical water losses, preventing soil drainage in northern wetlands during most of the summer despite low precipitation input^27^. Increasing rainfall^28^ and increased permafrost degradation can increase soil wetness in continuous permafrost regions^2^. Further permafrost degradation (e.g. ice-wedge melting) can increase hydrologic connectivity leading to increased lateral drainage of the landscape and subsequent soil drying^2,29^.

Given the importance of soil moisture in affecting the carbon balance of arctic ecosystems, and its links with snowmelt timing, in this study, we investigated the correlation between summer fluxes of CO_2_ (i.e., net ecosystem exchange (NEE), gross primary productivity (GPP) ecosystem respiration (ER)), ET, and environmental drivers such as soil moisture and snowmelt timing, while controlling for the other most important drivers of photosynthesis and respiration (i.e. solar radiation and air temperature). We expected earlier snowmelt to be correlated with larger ET and lower soil moisture, particularly during peak and late season, consistent with drying associated with a longer growing season. The lower soil moisture with earlier snowmelt should result in a negative correlation between snowmelt timing and GPP, particularly during the peak and late season (when we expect the most water stress), and in a positive correlation between snowmelt timing and ER during the entire growing season. This soil moisture limitation to plant productivity should result in lower net cumulative CO_2_ sequestration during the entire summer, because of lower plant productivity if these ecosystems are water-limited due to lower soil moisture with earlier snowmelt.

### Testing the impact of snowmelt timing on the carbon dynamics and hydrology of tundra ecosystems

**Figure 1:**
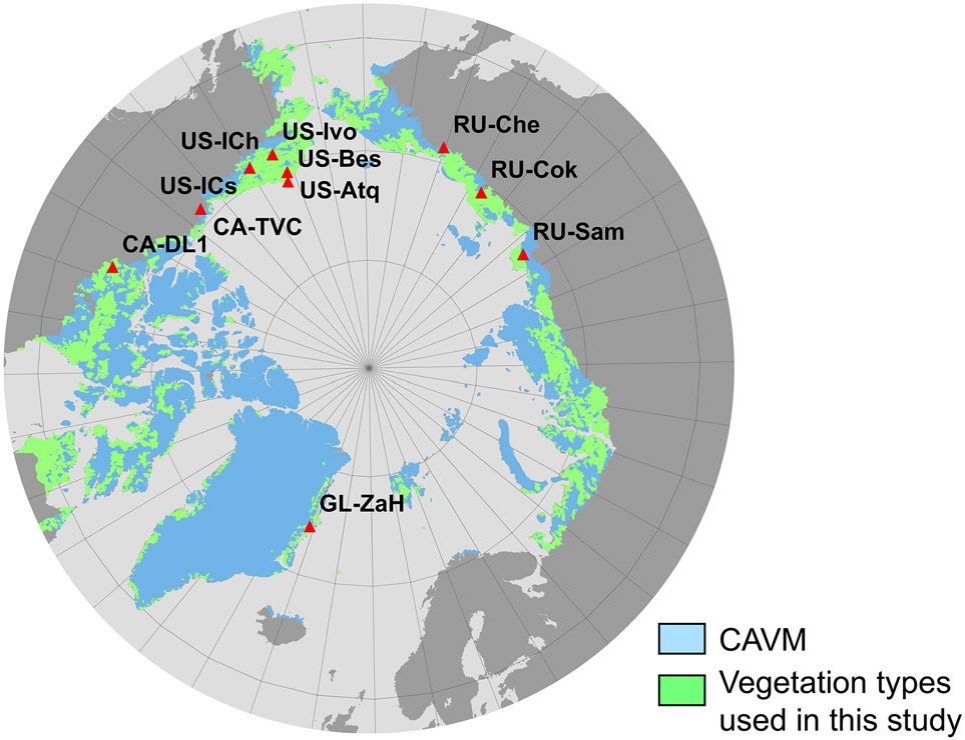
Study sites. Locations of the 11 eddy covariance flux tower sites used in this study. Light blue regions delineate the total Circumpolar Arctic Vegetation Map (CAVM), green regions delineate the subset of CAVM vegetation types represented in this study (including all the vegetation types listed in Table S1). This map was created using QGIS.org, 2020, QGIS 3.10. Geographic Information System User Guide. QGIS Association: https://docs.qgis.org/3.10/en/docs/user_manual/index.html. The dataset used in the map was the CAVM map: CAVM Team. 2003. Circumpolar Arctic Vegetation Map. (1:7,500,000 scale), Conservation of Arctic Flora and Fauna (CAFF) Map No. 1. U.S. Fish and Wildlife Service, Anchorage, Alaska. ISBN: 0-9767525-0-6, ISBN-13: 978-0-9767525-0-9.

The 11 sites were selected as among the longest-running tower sites in the circumpolar Arctic (including 6 to 19 years of fluxes per site and a total of 119 site-years of summer (June to August) eddy covariance CO_2_ flux data, Table S1). All sites lie in the zone of continuous permafrost. The sites are representative of dominant tundra vegetation classes (wetland, graminoids, and shrub tundra), together accounting for 31% of all tundra vegetation types (Fig. 1^30^ and Supplementary Information). Given the complex interactions among different variables (many covarying together), we used a variety of statistical analyses to identify the association between standardized anomalies of NEE, GPP, ER, and ET, and standardized anomalies of the main environmental controls during different periods of the summer corresponding to various stages in seasonal phenology (early season: June, peak season: July, and late season: August). We used a partial correlation analysis to identify if the timing of the snowmelt associates with anomalies of ET, soil moisture, NEE, GPP, ER, atmospheric vapor pressure deficit (VPD), or the Bowen ratio (the ratio between Sensible Heat (H) and Latent Heat (LE)) while statistically controlling for the main meteorological forcing such as air temperature and solar radiation (Methods). Identifying the correlation between ET (and the Bowen ratio) and snowmelt timing is a way to assess water limitation to ecosystems (in addition to testing their response to soil moisture changes), as H, and therefore, the Bowen ratio, are expected to increase with surface drying^31,32^. To identify the association between snowmelt timing, the main environmental variables (i.e., air temperature and solar radiation), and NEE, GPP, ER, and ET over time, we performed a maximum covariance analysis (MCA) on the monthly median standardized anomalies from 2004 to 2019 (a time period when data for most of the sites were available). MCA allowed us to find patterns in two space–time datasets that are highly correlated using a cross-covariance matrix^26^. We retained sites as the unit of variation (i.e., by estimating the standardized anomalies by site for each of the indicated variables, see “Methods”), hence the results of the MCA integrated the site level relationships between each of the variables over time). The goal of this analysis was to identify the most important environmental drivers associated with NEE, GPP, and ER across all the sites over time. MCA is particularly appropriate for this study as it can handle data with gaps and unequal lengths in the datasets. We also tested the relative importance of the abovementioned environmental drivers on the monthly median GPP, ER, and NEE using a linear mixed effect model, including site as a random effect to account for the site-to-site variability. The MCA and the mixed model analyses were conducted to test the relative importance of snowmelt and other variables at different times of the season. Finally, to evaluate the water balance at different times of the season, we estimated the difference between Potential Evapotranspiration (PET) and the actual ET, and the difference between precipitation (PPT) and ET for each of the sites, years, and months (e.g. June, July, and August). This study did not attempt to describe the long-term temporal changes in the anomalies of snowmelt and carbon fluxes, given the short data record available for some of the sites (i.e. less than 10 years, Table S1), but instead focused on understanding the association between environmental variables and the carbon balance at different times of the season. More details of these analyses are included in the Methods.

### Influence of snowmelt timing on NEE, GPP, ER, and hydrological status of tundra ecosystems

**Figure 2:**
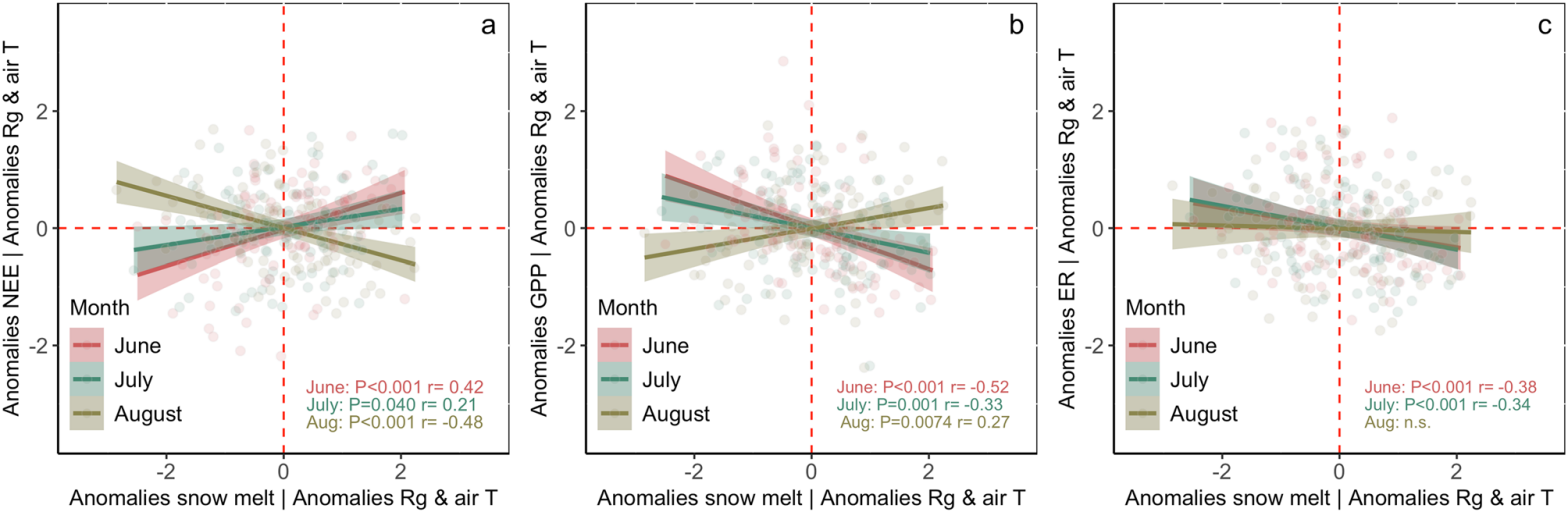
Relationships between the indicated median monthly anomalies using partial correlation analysis accounting for solar radiation and air temperature anomalies (retaining site as the unit of variation). Given that the interaction term between “month” and snowmelt timing was significant, we included the correlation coefficients and P of the regressions for each of the indicated months separately in each panel (also included in Table 1). Negative values indicate CO_2_ uptake and positive values CO_2_ release into the atmosphere, and shaded areas are 95% confidence intervals.

**Figure 3:**
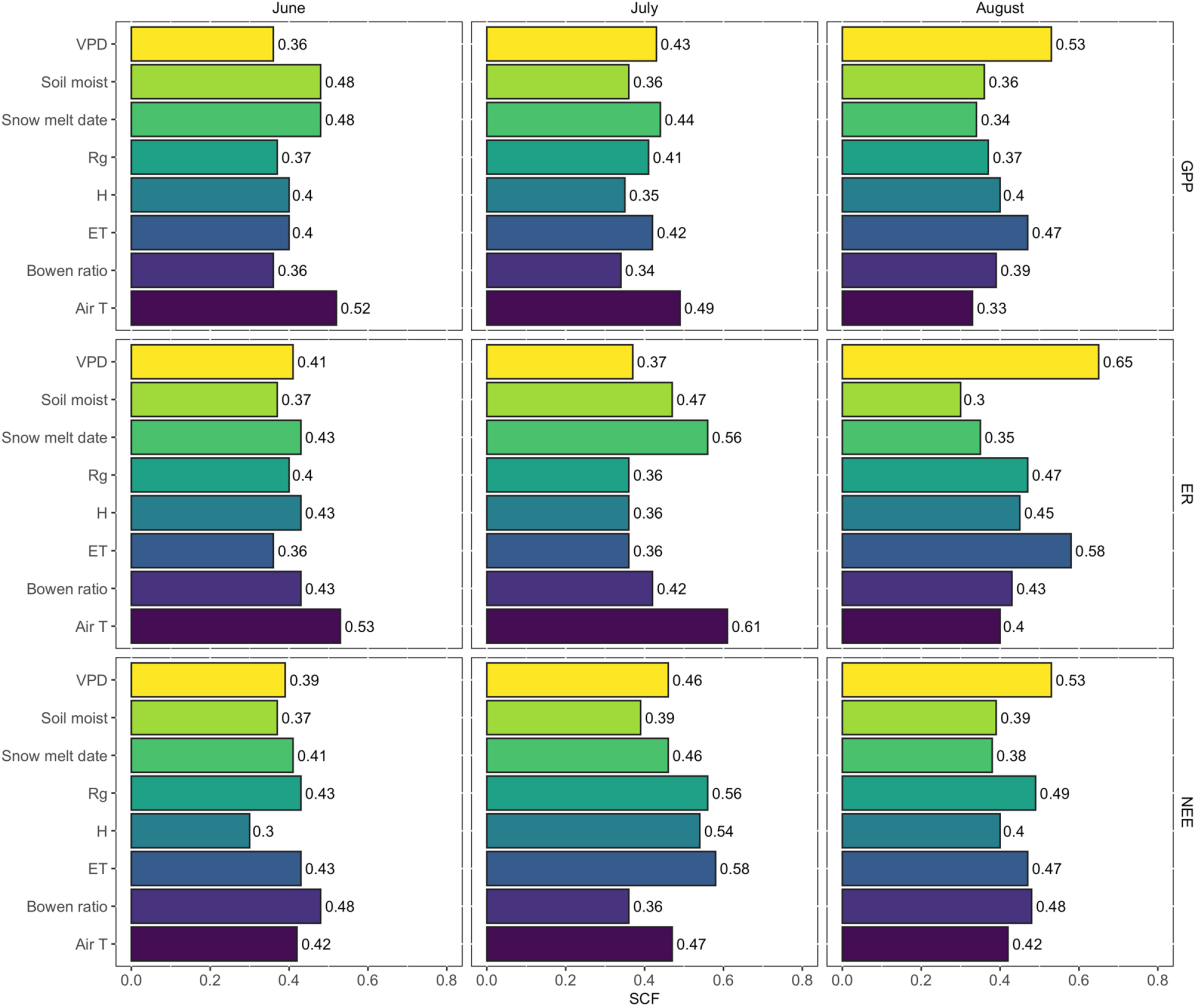
Squared covariance fraction (SCF) of each couple of the indicated variables for the maximum covariance analysis (MCA) of the monthly median anomalies of GPP, ER, and NEE in June, July, and August. The first pair of singular vectors are the phase-space directions when projected that have the largest possible cross-covariance. The singular vectors describe the patterns in the anomalies that are linearly correlated. A higher SCF indicates a stronger association over time between the indicated variables.

Once statistically controlling for solar radiation and air temperature (in the partial correlation analysis, see “Methods”), we observed a significant positive relationship between the snowmelt timing anomalies and NEE anomalies (i.e. earlier snowmelt was associated with a higher net CO_2_ sequestration) in June and July, but a negative correlation in August (Fig. 2a, Table 1). A significant relationship was also found between snowmelt date anomalies and GPP anomalies, with more positive GPP anomalies (i.e. higher plant productivity) with earlier snowmelt in June and July, and more negative GPP anomalies with earlier snowmelt in August (Fig. 2b, Table 1). Earlier snowmelt was associated with significantly higher ER in both June and July, but there was no significant relationship in August (Fig. 2c, Table 1), suggesting that the late-season correlation between NEE and snowmelt timing was mostly driven by the lower GPP and with earlier snowmelt in August. The MCA analysis showed that the anomalies in snowmelt timing had the highest squared covariance fraction (SCF) with the monthly median anomalies of GPP, NEE, and ER in June and July, and the lowest in August over the 2004–2019 period (Fig. 3). A similar result was observed in the linear mixed effect model, which showed a significant relationship between snowmelt date and GPP, and NEE, in all summer months, higher ${R}_{m}^{2}$ between the snowmelt date and GPP in June and July, and no significant relationship between snowmelt date and ER in August (Table S3). In late season, other environmental variables had a higher covariance with the GPP, NEE, and ER anomalies than the snowmelt timing (Fig. 3, Table S3).

**Table 1:** Significance (P) and Pearson’s correlation coefficient (r) of the relationships between the indicated monthly median standardized anomalies for June, July, and August retaining site as a unit of variation.

Regression model	Month	P	r
NEE ~ snow melt | Rg & air T	June	< 0.001	0.42
	July	0.040	0.21
	August	< 0.001	−0.48
GPP ~ snow melt | Rg & air T	June	< 0.001	−0.52
	July	0.001	−0.33
	August	0.0074	0.27
ER ~ snow melt | Rg & air T	June	< 0.001	−0.38
	July	< 0.001	−0.34
	August	–	–

Site was retained as the unit of variation by estimating the standardized anomalies by month and site for each of the indicated variables. The anomalies of the indicated variables were regressed with snow depth anomalies using a partial correlation accounting for the anomalies of solar radiation and air temperature, as shown in Fig. 2. The P and r were only included when the P < 0.1 (given that for P > 0.1 we assumed that r is not significantly different from zero), N = 284.

Our results are consistent with the discrepancy between the observed increase in the maxNDVI over the last four decades and the time-integrated (TI) NDVI which instead has plateaued in the last two decades and even decreased over the last 10 years in several northern arctic ecosystems^33^. TI-NDVI considers the length of the growing season and phenological variations^34^ and, therefore, better integrates vegetation development during the entire growing season. Moisture was shown to be important for the NDVI trends^33,35^. Given the potential water limitation to summer carbon uptake in northern ecosystems^12,23–25^, we tested if an earlier snowmelt was associated with a decrease in soil moisture, which would affect GPP and NEE. We only observed a significant correlation between soil moisture anomalies and snowmelt date anomalies in June (i.e. higher soil moisture with earlier snowmelt, Fig. S1a, Table S2), but no significant correlation in July and August (Fig. S1a, Table S2). The higher soil moisture with earlier snowmelt in June is consistent with surface inundation after snowmelt^36,37^ and earlier soil thawing resulting in higher soil moisture (i.e., soil moisture is low while soils are frozen). A similar result was observed for the ET anomalies. Higher ET with earlier snowmelt in June (Fig. S1b) could be the result of surface inundation after snowmelt^32^. The standardized NEE anomalies were significantly correlated with the soil moisture anomalies in each of the summer months (Fig. S1d, Table S2). However, the relationship between the GPP (and ER anomalies) and soil moisture anomalies was only significant in June (Fig. S1e,f, Table S2) suggesting an earlier activation of the vegetation with earlier soil thaw (and the associated higher soil moisture). A higher water loss from ET in early season (Fig. S1b) could have resulted in the drying of the surface moss layer with the progression of the summer, which would have been consistent with the observed lower GPP and the lower net CO_2_ sequestration with earlier snowmelt observed in August (Fig. 2a,b, Table 1). A potential moisture limitation to plant productivity might have been consistent also with the higher SCF of NEE, or GPP and VPD anomalies in August than in June and July (Fig. 3). However, no significant relationship between ET (or soil moisture) and snowmelt date anomalies was observed in July and August (Fig. S1a,b) contrary to what would be expected if drying occurred following earlier snowmelt. No significant relationship was found between VPD anomalies and snowmelt date anomalies in any of the summer months (P = 0.14 in a partial correlation considering air temperature and solar radiation anomalies). Finally, surface drying should result in an increase in the Bowen ratio anomalies with the progression of the summer, given that H increases with a decrease in water table and surface drying^32,38^. However, the Bowen ratio showed no correlation with the standardized snowmelt date anomalies in any of the summer months (Fig. S1c, Table S2), and presented similar values in all the summer months (Fig. S2a). The lack of correlation between the soil moisture, VPD, Bowen ratio, and snowmelt date anomalies suggests that an earlier snowmelt did not result in significant surface drying in the sites of this study. The median PET-ET and PPT-ET for all years and sites included in this analysis (Fig.S2 b,c) was slightly higher in August, similar to reports by others for the Russian arctic tundra^38,39^, further supporting a lack of soil moisture limitation in late season. Although these analyses do not consider runoff, which can be significant^21,26^, overall our results do not suggest that an earlier snowmelt resulted in a water stress that significantly limited plant productivity in these arctic ecosystems over continuous permafrost.

The correlation between the anomalies in the August GPP and snowmelt timing is consistent with earlier senescence in northern plant species (e.g. *Eriophorum vaginatum*, a dominant species across these tundra types) compared to southern species growing in the same location in a common garden experiment^40^. The phenotypic variation was shown to be persisting for decades^41^, and ecotypes may be unable to extend their effective growth period or take advantage of a longer growing season^40^. Several studies across different plant functional types have shown that once plant growth is initiated after the snowmelt in northern ecosystems, it continues only for a fixed number of days until the occurrence of senescence^42–44^. Therefore, the lower GPP in August with earlier snowmelt might not be linked to water limitation on photosynthesis later in the season, but rather to an earlier senescence arising from the endogenous rhythms of growth and senescence, that plant functional types living in these extreme conditions have developed over decades. On a broader scale, earlier senescence with an earlier start of the growing season after snowmelt in northern ecosystems is also consistent with an earlier spring zero-crossing date and an earlier autumn zero-crossing date of the mean detrended seasonal CO_2_ variations at Barrow, AK, USA (NOAA ESRL: https://www.esrl.noaa.gov/gmd/ccgg/obspack/) during 2013–2017 compared to 1980–1984^5^. The spring and autumn zero-crossing date is the time when the detrended seasonal CO_2_ variations intersect the zero line in spring and autumn respectively and can be used as an indicator for the start and end of the net CO_2_ uptake by vegetation^45,46^. On the other hand, NDVI measurements show both an earlier start of the season and a later end of season for 2008–2012 compared to 1982–1986^5^. The disagreement between the detrended seasonal atmospheric CO_2_ concentration showing an earlier autumn zero-crossing date and the NDVI measurements showing a later end of the season has been explained by the increase in respiration in the fall^20^. The disagreement between atmospheric CO_2_ concentration trends (showing an earlier autumn zero-crossing date), and NDVI (showing a later end of the season,^5^ may also be explained by the challenges in using NDVI as a proxy for plant productivity in these arctic systems. The relationship between NDVI and CO_2_ flux and plant productivity is highly variable and non-linear in arctic ecosystems^47^. While some arctic ecosystems have shown that NDVI was strongly correlated with GPP (explaining 75% of the variation in GPP^48^, other studies showed that NDVI was either not significantly correlated with GPP and NEE^49^ or was only able to explain a minor fraction (maximum of 25%) of the variation in NEE and GPP in some arctic tundra ecosystems after accounting for the seasonal variation^50,51^.

**Figure 4:**
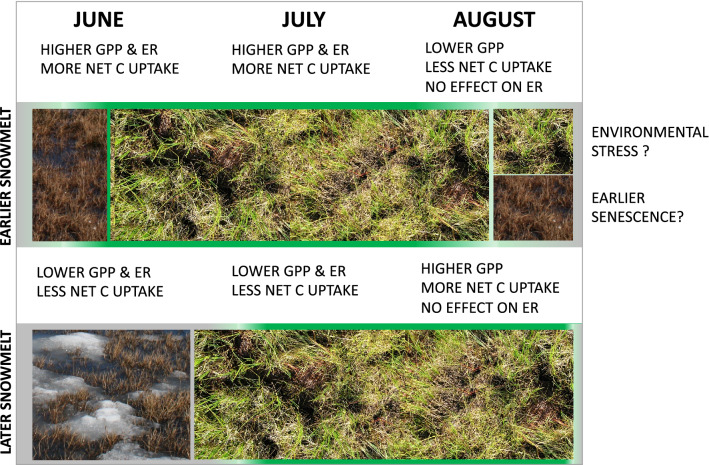
Schematic of the effect of earlier snowmelt on NEE, GPP, and ER at different times of the season. Earlier snowmelt results in an earlier activation of the vegetation, higher plant productivity, and higher net carbon uptake in June and July. This earlier activation could result in more carbon loss and lower plant productivity with earlier snowmelt in August, potentially related to either environmental stress, or to earlier senescence. Photo credit: Donatella Zona.

In conclusion, earlier snowmelt was associated with greater net CO_2_ uptake and higher GPP in early and peak seasons, but with less net CO_2_ uptake and lower GPP later in the summer, in the studied arctic tundra ecosystems. We did not find evidence of a late-season water limitation to GPP with earlier snowmelt. Although several hypotheses can be forwarded to explain the link between snowmelt and late season declines in plant productivity and carbon uptake, the current literature does not provide a definitive explanation (schematic Fig. 4). Future studies should investigate the potential interaction of different processes explaining the response of the carbon dynamics in the Arctic to earlier snowmelt and reconstruct the temporal changes in the carbon balance from these systems. The link between the long-term changes in the CO_2_ fluxes and NDVI in tundra ecosystems needs closer examination. Studies should investigate if higher NDVI is definitively associated with higher net CO_2_ uptake. Greening of the Arctic might not necessarily translate into more net CO_2_ uptake, as early and peak season carbon gains might be offset by a late-season CO_2_ loss, and respiration might counterbalance the increase in plant productivity. A better understanding of the processes driving these temporal changes is a fundamental step in advancing our prediction of the response of the arctic CO_2_ balance to changing climate.

## Materials and methods

### Site description

A total of 11 eddy covariance flux tower sites across the Arctic were used in this study, where each site had at least six summers of flux data available (SI, Table S1). Ecosystem-scale CO_2_ fluxes were estimated using the eddy covariance method^52–54^. Details pertaining to the sites, data processing, and gap-filling are provided in the SI Appendix, Table S1. All sites are located in continuous permafrost tundra regions. Vegetation at the tower sites, the instruments used to measure fluxes, the average environmental conditions at each site, the datasets used in this study for each site, and the references describing the sites are indicated in SI Appendix, Table S1. As shown in Fig. S2, the Bowen ratio reported for this study showed similar values to what previously reported during the growing season months in the Arctic (from 3.9 in a dry heath to 1.6 in a wet fen in Greenland^31^; 0.83^38^ to 0.20–0.25 in two Siberian Arctic sites^39^; and 0.51–1.69 in a moist-tussock tundra in Alaska^32^.

To estimate the standardized anomalies in the soil moisture we selected the most consistent depths and sensors (i.e., the same sensor available for the entire time period in each site, or sensors at the most similar depths in each site and across sites when data from the same sensor was not available due to instrument failure). The number of sensors and the soil depths in each of the sites used for all the analyses were: (CA-DL1: N = 2, one in a wet location and one in a dry location (both at −10 cm depth); US-Atq: N = 1 (2010–2019) (−10 cm depth); US-Ivo: N = 4 at −5 cm depth; US-Bes: N = 2 (2 diagonally inserted at 0–10 cm); RU-Che: N = 2 (−8 cm and −16 cm depth); RU-Sam: N = 5 (at −5, −14, in rims at −5, −12, −15 cm depths in the center of ice-wedge polygons); US-ICs: N = 2 (at −2.5 cm depth); GL-ZaH: N = 2 (2000–2004 vertical 0–6 cm and from 2005 onward are at two horizontal depths : −5 cm, −10 cm); CA-TVC: included one sensor inserted horizontally at −20 cm depth. More details on the temporal coverage of the soil moisture data from each site are included in Supplementary Table S1.

The R package ‘Evapotranspiration’ (Version 1.15^55^) was used to estimate the daily aggregated Priestley-Taylor potential evaporation^56,57^ at each of the study sites, then summed into monthly totals. We subtracted monthly total actual ET measured with eddy covariance at the respective sites to estimate the PET-ET shown in Fig. S2b. Raster files of monthly precipitation accumulation were acquired for the months of June–August from TerraClimate^58^ over the years 1959–2019. Precipitation data was then extracted for the Eddy Covariance tower coordinates using the terra package^59^ in R^60^ to estimate monthly total precipitation for June, July, and August for each of the sites (Tables S1). We did not use the precipitation collected by the meteorological sensors installed in the tower sites given large gaps in the site-level dataset. The other environmental variables used in this study were collected at the tower sites. The median difference between the total precipitation and the total ET in each site was estimated to evaluate the PPT-ET at each study site during each time of the season (June, July, and August as shown in Fig. S2c). The median was used as it is less affected by outliers.

### Statistical analysis

#### Site-level data

For the analyses performed in this study, we separated the data into different times of the season (early season: June, peak season: July, late season: August), given that some of the environmental controls could be very different given the distinct stages of vegetation development. A partial correlation analysis was carried out to identify the correlation between the monthly median standardized anomalies (the ratio between the anomalies and the climatological standard deviation) of NEE, GPP, ER, ET, snowmelt date, and other environmental variables (most of which covary). The standardized monthly median anomalies were estimated by calculating the monthly median of the standardized weekly anomalies. The standardized weekly anomalies were estimated by subtracting the observed values (for each week, year, and site) from the respective average values (for each week, and site and for the entire period available in each site), and then standardized by dividing the anomalies by the standard deviation for each week, and site for the entire period available in each site. The standardized median monthly anomalies were used instead of weekly values, given that the median is less affected by outliers. The monthly scale was used for all the variables included in these analyses as the most appropriate temporal scale to identify the importance of the variability in soil moisture on CO_2_ fluxes (given that soil moisture does not change much at the hourly and weekly scale at these tundra sites).

The NEE, GPP, ER data used in these analyses were gap-filled using standard methodologies as described in the Supplementary Information. The partial correlations tested the relationships between the standardized snowmelt date anomalies and the monthly median standardized anomalies of NEE, GPP, ER, ET, VPD, and soil moisture, retaining site as the unit of variation (while controlling for solar radiation and air temperature anomalies as the main controls on carbon fluxes). We also tested if the inclusion of a given site within a linear mixed model changed the results of the correlation analysis between the anomalies. To this purpose, linear mixed effects models (nlme package in R^60^) were used to test the significance of the correlation between the above-mentioned anomalies, by including “site” as categorical random effects to account for pseudo-replication due to the different sites measured in different years. Model performance was evaluated based on the Akaike information criterion (AIC) values, on the marginal coefficient of determination (${R}_{m}^{2}$ similar to the explanatory power of the linear models) for generalized mixed-effects models as output by the “r.squaredGLMM” function within the “MuMIn” package in R^61,62^. These analyses are included in Table 1, Table S2, and Table S3.

To maximize the dataset for each analysis we included all available time periods for the variables regressed in Fig. 2, but only selected the 2004–2019 period for the Maximum Covariance Analysis (MCA) to include a time period where most sites had data available. The MCA was performed on two fields (i.e., anomalies in NEE, GPP, and ER and anomalies in the environmental drivers, see Fig. 3); the columns of the two fields are spatial locations (each site was retained as a unit of variation of this analysis) and rows are temporal measurements. Site was retained as the unit of variation by estimating the median standardized anomalies by year, week, and site for each of the indicated variables, and then by estimating a monthly median from these weekly anomalies. The first pair of singular vectors are the phase-space directions when projected that have the largest possible cross-covariance. The singular vectors describe the patterns in the anomalies that are linearly correlated. We used the time series of the first singular value decomposition (SVD) mode to visualize the parts of the datasets that vary together and reported the squared covariance fraction (SCF) of the MCA in Fig. 3. Given the limited length of the dataset we did not discuss the long-term changes in the reported anomalies. However, the MCA allowed us to evaluate the influence of snowmelt timing on the carbon balance over time at different times of the growing season, and compare its relative importance to other variables. All analyses were carried out in R^60^.

## Supplementary Information

Supplementary Information.

## Data Availability

The eddy covariance data from RU-Che, RU-Cok, and GL-ZaH (previously named DK-ZaH), CA-DL1, were obtained from the European Fluxes Database (http://www.europe-fluxdata.eu/home), from the Ameriflux Database (http://ameriflux.lbl.gov/), with some updated versions provided directly by the principal investigators of each site (e.g. the data from GL-ZaH are also available on: https://data.g-e-m.dk). The data from US-ICh and US-ICs are stored in the http://aon.iab.uaf.edu/data_access. US-Bes, US-Atq, US-Ivo are stored in the Arctic Data Center (Donatella Zona. 2021. Greenhouse gas flux measurements at the zero curtain, North Slope, Alaska, 2012–2021. Arctic Data Center. doi:10.18739/A2ZG6G80B.). The R code for the MCA analysis is available in the webpage: https://edoras.sdsu.edu/~babailey/ (https://edoras.sdsu.edu/~babailey/mcaplot/).

## References

[1] Overland, J. E., *et al.**The NOAA Arctic Report Card, Surface Air Temperature*. https://arctic.noaa.gov/Report-Card/Report-Card2019/ArtMID/7916/ArticleID/835/Surface-Air-Temperature. (2019).

[2] Liljedahl AK (2016). Pan-Arctic ice-wedge degradation in warming permafrost and its influence on tundra hydrology. Nat. Geosci..

[3] Mudryk LR, Kushner PJ, Derksen C, Thackeray C (2017). Snow cover response to temperature in observational and climate model ensembles. Geophys. Res. Lett..

[4] Mudryk, L., Brown, R., Derksen C., Luojus K., Decharme B., & Helfrich S. Terrestrial snow cover. In *Arctic Report Card 2019.* (Richter-Menge, J., Druckenmiller, M. L., Jeffries, M. Eds.). https://www.arctic.noaa.gov/Report-Card. (2019).

[5] Piao S (2020). Characteristics, drivers and feedbacks of global greening. Nat. Rev. Earth Environ..

[6] Lucht W (2002). Climatic control of the high- latitude vegetation greening trend and Pinatubo effect. Science.

[7] Berner LT (2020). Summer warming explains widespread but not uniform greening in the Arctic tundra biome. Nat. Commun..

[8] Myers-Smith IH (2020). Complexity revealed in the greening of the Arctic. Nat. Clim. Change.

[9] Forbes BC, Fauria MM, Zetterberg P (2010). Russian Arctic warming and ‘greening’ are closely tracked by tundra shrub willows. Glob. Change Biol..

[10] Lara MJ, Nitze I, Grosse G, Martin P, McGuire AD (2018). Reduced arctic tundra productivity linked with landform and climate change interactions. Sci. Rep..

[11] Miles VV, Esau I (2016). Spatial heterogeneity of greening and browning between and within bioclimatic zones in northern West Siberia. Environ. Res. Lett..

[12] Gonsamo A, Ter-Mikaelian MT, Chen JM, Chen J (2019). Does earlier and increased spring plant growth lead to reduced summer soil moisture and plant growth on landscapes typical of tundra–taiga interface?. Remote Sens..

[13] Gamm CM (2018). Declining growth of deciduous shrubs in the warming climate of continental western Greenland. J. Ecol..

[14] Bruhwiler L, Parmentier F-JW, Crill P, Leonard M, Palmer PI (2021). The Arctic carbon cycle and its response to changing climate. Curr. Clim. Change Rep..

[15] Humphreys ER, Lafleur PM (2011). Does earlier snowmelt lead to greater CO_2_ sequestration in two low Arctic tundra ecosystems?. Geophys. Res. Lett..

[16] Parmentier FJW (2011). Spatial and temporal dynamics in eddy covariance observations of methane fluxes at a tundra site in northeastern Siberia. J. Geophys. Res. Biogeosci..

[17] Lund M (2012). Trends in CO_2_ exchange in a high Arctic tundra heath, 2000–2010. J. Geophys. Res. Biogeosci..

[18] Ueyama M (2013). Growing season and spatial variations of carbon fluxes of Arctic and boreal ecosystems in Alaska (USA). Ecol. Appl..

[19] López-Blanco E (2020). Multi-year data-model evaluation reveals the importance of nutrient availability over climate in arctic ecosystem C dynamics. Environ. Res. Lett..

[20] Piao S (2008). Net carbon dioxide losses of northern ecosystems in response to autumn warming. Nature.

[21] Liljedahl AK, Hinzman LD, Kane DL, Oechel WC, Tweedie CE, Zona D (2017). Tundra water budget and implications of precipitation underestimation. Water Resour. Res..

[22] Park T (2016). Changes in growing season duration and productivity of northern vegetation inferred from long-term remote sensing data. Environ. Res. Lett..

[23] Parida BR, Buermann W (2014). Increasing summer drying in North American ecosystems in response to longer nonfrozen periods. Geophys. Res. Lett..

[24] Angert A (2005). Drier summers cancel out the CO_2_ uptake enhancement induced by warmer springs. Proc. Natl. Acad. Sci. U.S.A..

[25] Buermann W (2018). Widespread seasonal compensation effects of spring warming on northern plant productivity. Nature.

[26] Lian X (2020). Summer soil drying exacerbated by earlier spring greening of northern vegetation. Sci. Adv..

[27] Rouse WR (2000). The energy and water balance of high-latitude wetlands: Controls and extrapolation. Glob. Change Biol..

[28] Zhang X (2013). On the variation of regional CO_2_ exchange over temperate and boreal North America. Global Biogeochem. Cycles.

[29] Christensen TR (2021). Multiple ecosystem effects of extreme weather events in the Arctic. Ecosystems.

[30] Walker DA (2005). The circumpolar Arctic vegetation map. J. Veg. Sci..

[31] Stiegler C, Lund M, Christensen TR, Mastepanov M, Lindroth A (2016). Two years with extreme and little snowfall: Effects on energy partitioning and surface energy exchange in a high-Arctic tundra ecosystem. Cryosphere.

[32] Vourlitis GL, Oechel WC (1997). Landscape-scale CO_2_ H_2_O vapour and energy flux of moist-wet coastal tundra ecosystems over two growing seasons. J. Ecol..

[33] Bhatt US (2021). Climate drivers of Arctic tundra variability and change using an indicators framework. Environ. Res. Lett..

[34] Tucker CJ, Sellers PJ (1986). Satellite remote sensing of primary production. Int. J. Remote Sens..

[35] Arndt KA (2019). Arctic greening associated with lengthening growing seasons in Northern Alaska. Environ. Res. Lett..

[36] Bowling LC, Kane DL, Gieck RE, Hinzman LD, Lettenmaier DP (2003). The role of surface storage in a low-gradient Arctic watershed. Water Resour. Res..

[37] Woo M-K, Young KL, Brown L (2006). High Arctic patchy wetlands: Hydrologic variability and their sustainability. Phys. Geogr..

[38] Goeckede M (2017). Shifted energy fluxes, increased Bowen ratios, and reduced thaw depths linked with drainage-induced changes in permafrost ecosystem structure. Cryosphere.

[39] Runkle BRK, Wille C, Gažovič M, Wilmking M, Kutzbach L (2014). The surface energy balance and its drivers in a boreal peatland fen of northwestern Russia. J. Hydrol..

[40] Parker TC, Tang J, Clark MB, Moody MM, Fetcher N (2017). Ecotypic differences in the phenology of the tundra species *Eriophorum vaginatum* reflect sites of origin. Ecol. Evol..

[41] Souther S, Fetcher N, Fowler Z, Shaver GR, McGraw JB (2014). Ecotypic differentiation in photosynthesis and growth of *Eriophorum vaginatum* along a latitudinal gradient in the Arctic tundra. Botany.

[42] Bjorkman AD, Elmendorf SC, Beamish AL, Vellend M, Henry GHR (2015). Contrasting effects of warming and increased snowfall on Arctic tundra plant phenology over the past two decades. Global Change Biol..

[43] Rosa RK (2015). Plant phenological responses to a long-term experimental extension of growing season and soil warming in the tussock tundra of Alaska. Glob. Change Biol..

[44] Semenchuk PR, Gillespie MAK, Rumpf SB, Baggesen N, Elberling B, Cooper EJ (2016). High Arctic plant phenology is determined by snowmelt patterns but duration of phenological periods is fixed: an example of periodicity. Environ. Res. Lett..

[45] Keeling CD, Chin JFS, Whorf TP (1996). Increased activity of northern vegetation inferred from atmospheric CO_2_ measurements. Nature.

[46] Piao S (2017). Weakening temperature control on the interannual variations of spring carbon uptake across northern lands. Nat. Clim. Change.

[47] Beamish A (2020). Recent trends and remaining challenges for optical remote sensing of Arctic tundra vegetation: A review and outlook. Remote Sens. Environ..

[48] Street LE, Shaver GR, Williams M, Van Wijk MT (2007). What is the relationship between changes in canopy leaf area and changes in photosynthetic CO_2_ flux in arctic ecosystems?. J. Ecol..

[49] Zona D, Oechel WC, Peterson KM, Clements RJ, Paw UKT, Ustin SL (2010). Characterization of the carbon fluxes of a vegetated drained lake basin chronosequence on the Alaskan Arctic Coastal Plain. Glob. Change Biol..

[50] La Puma IP, Philippi TE, Oberbauer SF (2007). Relating NDVI to ecosystem CO_2_ exchange patterns in response to season length and soil warming manipulations in arctic Alaska. Remote Sens. Environ..

[51] Olivas PC, Oberbauer SF, Tweedie C, Oechel WC, Lin D, Kuchy A (2011). Effects of fine-scale topography on CO_2_ flux components of Alaskan coastal plain tundra: Response to contrasting growing seasons. Arct. Antarct. Alp. Res..

[52] Burba GG, McDermitt DK, Grelle A, Anderson DJ, Xu L (2008). Addressing the influence of instrument surface heat exchange on the measurements of CO_2_ flux from open-path gas analyzers. Glob. Change Biol..

[53] Burba G (2012). Calculating CO_2_ and H_2_O eddy covariance fluxes from an enclosed gas analyzer using an instantaneous mixing ratio. Glob. Change Biol..

[54] Burba, G. Eddy covariance method for scientific, industrial, agricultural and regulatory applications: A field book on measuring ecosystem gas exchange and areal emission rates. in *LI-COR Biosciences*. 331. ISBN: 978-0-61576827-4. (2013).

[55] Guo D, Westra S, Maier HR (2016). An R package for modelling actual, potential and reference evapotranspiration. Environ. Modell. Softw..

[56] McMahon TA, Peel MC, Lowe L, Srikanthan R, McVicar TR (2013). Estimating actual, potential, reference crop and pan evaporation using standard meteorological data: A pragmatic synthesis. Hydrol. Earth Syst. Sci..

[57] Priestley C, Taylor R (1972). On the assessment of surface heat flux and evaporation using largescale parameters. Mon. Weather Rev..

[58] Abatzoglou JT, Dobrowski SZ, Parks SA, Hegewisch KC (2018). TerraClimate, a high-resolution global dataset of monthly climate and climatic water balance from 1958–2015. Sci. Data.

[59] Hijmans, R. J. *terra: Spatial Data Analysis. R Package Version 1.1-4*. https://CRAN.R-project.org/package=terra (2021).

[60] R Core Development Team. *R: A Language and Environment for Statistical Computing v. 4.0.2*. (R Foundation for Statistical Computing, 2020).

[61] Johnson PCD (2014). Extension of Nakagawa & Schielzeth's R2GLMM to random slopes models. Methods Ecol. Evol..

[62] Nakagawa S, Schielzeth H (2013). A general and simple method for obtaining R^2^ from generalized linear mixed-effects models. Methods Ecol. Evol..

