# Supplementary material for: Earlier snowmelt may lead to late season declines in plant productivity and carbon sequestration in Arctic tundra ecosystems
Source: Sci Rep. 2022 Mar 21;12:3986. doi: 10.1038/s41598-022-07561-1 (PMC8938415; doi:10.1038/s41598-022-07561-1)
Supplement: Supplementary file 1 — Supplementary Information. [file 41598_2022_7561_MOESM1_ESM.docx]

**Supplementary information**

**Study sites**

Eleven sites (Table S1) were selected representing Arctic tundra ecosystems on continuous permafrost with at least six years of flux data; ten of these sites (all of the sites except RU-Cok, which did not have soil moisture available) were used to test the correlation of soil moisture anomalies with NEE, GPP, and ER anomalies as detailed in the Methods. Flux observations were obtained using the eddy covariance technique [54] to determine land-atmosphere CO_2_ exchange at the ecosystem scale. The vegetation at all of the sites was classified according to [30], with US-Bes classified as W1 (sedge/grass moss wetland, wet coastal plain dominated by sedges, grasses, and mosses), US-Atq, RU-Sam, and RU-Cok classified as W2 (sedge moss/dwarf-shrub wetland, tundra dominated by sedges, grasses, mosses, and some dwarf shrubs < 40 cm tall), RU-Che as W3 (sedge, moss, low-shrub wetland, dominated by sedges and low shrubs > 40 cm tall), US-Ivo and RU-Cok, and US-ICt as G4 (tussock-sedge dwarf-shrub, moss tundra), GL-ZaH as P2 (prostrate/hemiprostrate dwarf-shrub tundra, moist to dry tundra dominated by prostrate and hemiprostrate shrubs < 15 cm tall, mosses, rushes, forbs, and lichens), and CA-DL1 and CA-TVC as S1 (erect dwarf-shrub tundra dominated by erect dwarf-shrubs, mostly < 40 cm tall, mosses, and lichens). The land cover types in US-Bes, RU-Che, and US-Atq include vegetation and landscape characteristics commonly found in pan-Arctic wetland categories [30]; US-Ivo, and US-ICt represent the dominant tundra vegetation types in Alaska (e.g., the subzone including US-Ivo and US-ICt accounts for about 83% of the landscape of the North Slope of Alaska [63]. The vegetation type at GL-ZaH is representative of approximately 10% of the high and middle Arctic [17]. The vegetation types of all of the above-mentioned sites combined represent 31% of all the vegetation types across the entire Arctic [30].

**Eddy covariance data processing**

Details on the site-level data processing are provided in the references listed in Table S1. For the sites where CO_2_ fluxes were measured using open-path analyzers (e.g., US-Bes, US-Atq, US-Ivo, and US-ICt), we included a description of corrections detailed in 64Oechel et al., 2014, and 52Burba et al., 2008, and examined the resulting influence on the fluxes. All of the data were processed using a common harmonized data processing consistent with the Ameriflux/Euroflux protocols. The half-hourly fluxes from US-Bes, US-Atq, US-Ivo were calculated using the EddyPro software v. 5.1.0 (LI-COR, Lincoln, NE, USA), as described in [65]. Open-path surface heating correction [52] was applied to the hourly CO_2_ fluxes following the procedure described in [64], with nearly-identical adjustment at the various sites. The effect of the adjustment was small, with slopes from 1.022 to 1.033 and offsets from 0.011 to 0.012 mg CO_2_ m^-1^ s^-1^, with adjusted correction being slightly smaller in magnitude in comparison to the original one. The effect of the correction was also quite small, with slopes from 1.022 to 1.034 and offset from -0.009 to -0.011 mg CO_2_ m^-1^ s^-1^, in comparison to the original uncorrected fluxes. The correction slightly reduced the CO_2_ uptake and increased the CO_2_ releases in comparison with uncorrected values, providing a small but consistent impact as expected at these primarily cold ecosystems. The time series of eddy covariance data from US-ICt, Alaska is described in [66]. At this site, an open-path LI-COR 7500 IRGA was installed in 2008, and to evaluate the influence of surface heating on the open-path LI-COR 7500 IRGA [52], an enclosed-path analyzer was installed in 2013. As the enclosed path LI-7200 is not subject to surface heating issues, the comparison of the open-path and enclosed path data enables evaluating the impact of heating on the CO_2_ fluxes during the cold period [67]. We found that overall, before correction, the LI-7500 showed slight uptake of CO_2_ during the winter, while the LI-7200 showed release [66]. Upon application of the heating correction [52], the estimated fluxes from the LI-7500 and LI-7200 analyzers generally agreed well. The largest absolute magnitude of the correction, observed in the coldest periods of winter, was still quite small, on the order of ~5.4×10^-6^ gC-CO_2_ m^-2^ s^-1^, and was in line with, or slightly below that discussed in 64Oechel et al., 2014, 52Burba et al., 2008. This correction was negligible in summer, which is the only time period included in this study.

**Gap Filling of the eddy covariance flux data**

The half-hourly CO_2_ eddy covariance flux data were gapfilled using the standard methodology of Ameriflux/Euroflux for all sites. As described in detail in [68], the marginal distribution sampling (MDS) method was used for the gapfilling. This method selects meteorological conditions physically and temporally similar to the ones for the missing data point(s), using solar radiation, air temperature, and VPD to the available variables. For the US-ICh and US-ICs the mean diurnal variation method of gap-filling, which has the advantage of not relying on environmental variables [66]. The more recent data have even better coverage in terms of gaps for power issues, equipment maintenance etc. For some of the Alaskan sites (US-Bes, and US-Atq) when this standard gap-filling methodology was not performing well due to large gaps in the data, particularly during the fall and winter periods, we used an alternative neural network approach run over several consecutive years [67, 69]. The neural network approach includes air and soil temperature, solar radiation, vapor pressure deficit (VPD), relative humidity (RH), ‘fuzzy’ datasets representing seasons, and an offset node [69]. The network had four hidden nodes and sigmoid transfer functions applied to both layers. We trained and fit the network 100 times and used the median value for each missing half-hour data period to fill gaps. We cross-compared the neural network and standard gap-filling methodologies which showed a good agreement when the data coverage was > 70% (i.e. June – August) and revealed large deviations in the standard methodology when data coverage was < 35% (i.e., September - May), indicating that the standard gap-filling method did not properly perform in presence of large data gaps. Precisely, we compared the daily Net Ecosystem Exchange, NEE from US-Bes during June 2009 to May 2010 estimated using both the standard Ameriflux/Fluxnet gap-filling and the neural network (Artificial Neural Network, ANN) gap-filling. The standard Ameriflux/Fluxnet gap-filling performed well when applied to data from periods with good coverage (> 70%) but the ANN was used with data with poor coverage (< 35%) during the cold season. Orthogonal regression results between the ANN and the standard Ameriflux/Fluxnet gap-filling were y = 1.13x + 0.02, P < 0.001 and Pearson’s r = 0.98 for the subset with good data coverage, whereas results from the period with poor data coverage showed lower correlation between the two methods: y = 2.71x – 0.16, P = 0.11 and Pearson’s r = 0.44.

The overall percentage data coverage for the June-August period for the entire period used in this study for each of the sites was: 67% for RU-Sam, 65% for CA-TVC, 95% for US-ICs, and US-ICh [66], 59% for US-Atq, 58% for US-Bes, 49% for US-Ivo, 65% for RU-Cok, 68% for RU-Che, 73% for CA-DL1, 53% for GE-ZaH.

**Snowmelt date across the eddy covariance tower sites**

A combined MODIS snow cover product (collection 5 MOD10A1/MCD10A1, [70] was used to estimate the dates of the initiation of the snowmelt. This method was selected after investigating the MODIS product against in situ air and soil temperature measurements, pictures from a local camera, and snow depth measurements collected with a SR50A-L Sonic Ranging Sensor (Campbell Scientific, Inc., Logan, UT, USA) in US-Ivo, and validated by comparison with the snowmelt dates estimated from direct observations in US-Ivo, US-Bes, US-ICt, and DK-ZaH, showing a very good comparison (y=1.13x, r=0.96 and p-value<0.001). The use of a MODIS snow cover product assured a spatial resolution (~500 m) appropriate for the eddy covariance tower measurements, consistency among all sites and years, and allowed extending the record for snowmelt date to years when no field data were available. The combined MODIS product used a maximum value approach on the daily snow cover extent estimates to alleviate low bias [71]. Yearly snowmelt date is registered as the first date on which fractional snow cover reached a 5% snow cover extent threshold. For every location, a daily maximum value composite time series was generated using a circular window of 500 m around the location of the tower. This circular window was centered at each of the eddy covariance sites; the fractional snow cover of each particular pixel within the circle was used to weigh the final value. Pixel values were extracted from the area weighted using a fraction of the circular window. Extractions were done using Google Earth Engine which uses the original sinusoidal grid (without regridding and interpolating). At high latitudes pixels are elongated over larger areas due to view angles. To account for projection issues the use of a region of interest rather than the single pixel location value is a conservative approach. The algorithm we used selected a circle of 500m diameter (centered in each of the tower location) and performed a weighted mean. Given the skewed pixels this methodology is more accurate than using the pixels- as the pixels get stretched in the poles (the region of interest allows selecting only the section of the tiles that matches 500m diameter).

**Additional support for this study**

Funding for E.E. was provided by the NSF Arctic Observatory Network (award numbers 0732594, 1107892, and 1503912). A.K.L. acknowledges support from NSF-OPP 1722572. H.K. acknowledges support from the NSF Macrosystems Biology program (award EF-1065029) and the Belgian Science Policy Office (contract BR/175/A3/COBECORE). Funding for J.D.W. was provided through the NASA New Investigator Program (NNH17ZDA001N-NIP) and a grant through the Gordon and Betty Moore Foundation. G.B. acknowledges both LI-COR Biosciences and the University of Nebraska for allowing the time to work on the manuscript. X.X. acknowledges the financial support from the Department of Energy – Oak Ridge National Laboratory (4000145166). This work partially used the Extreme Science and Engineering Discovery Environment (XSEDE), which is supported by National Science Foundation grant number ACI-1053575. X.X. appreciated assistance from Dr. Xiaochun Zhang on processing MODIS data in comparison with modeled output. Data from Zackenberg was provided by the Greenland Ecosystem Monitoring (GEM) program. Data collection at Ru-Sam was supported by the Cluster of Excellence “CliSAP” (EXC177), University of Hamburg, funded by the German Research Foundation (DFG) and the Helmholtz Association of German Research Centres (grant VH-NG-821 to T.S.) and the Helmholtz infrastructure funding ACROSS (Advanced Remote Sensing - Ground Truth Demo and Test Facilities). The work at Trail Valley Creek was funded through the Canada Research Chairs Program, the Canada Foundation for Innovation, the Polar Continental Shelf Program. CA-DL1 and CA-TVC were supported by the Natural Science and Engineering Council of Canada Discovery Grant program through grants awarded to P.M.L., E.R.H. and O.S. and the Natural Science and Engineering Council of Canada Discovery Grant program through grants awarded to P.M.L., E.R.H., and O.S.

A portion of the analysis included in this paper was performed at the Jet Propulsion Laboratory, California Institute of Technology, under contract with the National Aeronautics and Space Administration. We would like to thank the Global Change Research Group at San Diego State University, in particular, Patrick Murphy and UMIAQ and UIC for logistical support and for the help in the field, and John Crockett for the help with editing the manuscript.

**Supporting Tables**

**Table S1** Eddy covariance data used in this study. Indicated are the locations, the years for which data for each of the sites are included in this study, the eddy covariance CO_2_ flux instruments used, the vegetation type classification according to 30Walker et al., 2005 the average summer soil moisture and standard deviations (%) in the sensors available for each site (depth and number of sensors are indicated as footnote to the table below), and the main references describing the site.

| SITE | COUNTRY | | COORDINATE | Flux YEARS  (Soil moisture  if different) | EDDY COVARIANCE  CO_2_ flux INSTRUMENT | VEG TYPE | Soil moisture June-Aug**  (%) ± sd (se) | REF |  |
| --- | --- | --- | --- | --- | --- | --- | --- | --- | --- |
| US-Bes | | USA | 71.280881N,  156.596467W | 2005-2011  2014-2019  (2006-2010  2012-2019) | 2005-2013 Open path LI-7500  2013-2019  Closed path LGR-FGGA-24EP | W1 sedge/grass moss wetland | 59 ± 9 (3) | [65, 67] |  |
| US-Atq | | USA | 70.4696228N,  157.4089471W | 2004-2008  2011-2018  (2010-2018) | 2004-2008 Open path LI-7500  2011-2013  Enclosed path LI-7200  2013-2018  Closed path LGR-FGGA-24EP | W2 sedge moss/dwarf-shrub wetland | 55 ±9 (3) | [65, 67] |  |
| US-Ivo | | USA | 68.4864 N, 155.7502 W (2004-2007)  68.4805 N, 155.7568 W  (2014-) | 2004-2007  2013-2018  (2014-2019) | 2004-2008 Open path LI-7500  2013-2018  Enclosed path LI-7200 | G4 tussock-sedge, dwarf-shrub, moss tundra | 58 ± 6 (3) | [65, 67] |  |
| US-ICh | | USA | 68.607 N, 149.296 W | 2008-2019 | Open path LI7500; 2013 -2019 Open path  LI7500A | G4 tussock-sedge, dwarf-shrub, moss tundra (Dryasintegrifolia, lichen,Carexspp., dwarf evergreen, and deciduous shrub) | 67 ± 5 (2) | [66, 72, 73] |  |
| US-ICs | | USA | 68.606 N, 149.311 W | 2008-2019 | Open path LI7500; 2013 -2019 Open path  LI7500A | G4 tussock-sedge, dwarf-shrub, moss tundra (variety of Carex species, Eriophorumangustifo lium,and dwarf deciduous shrubs such asBetula nana,Salixspp, and mosse) | 67 ± 5 (2) | [66, 72, 73] |  |
| GL-ZaH (DK-ZaH) | | Greenland | 74.4732 N  20.5503W | 2000-2019 | 2000-2007 Closed path LI6262  2007-2016  Closed path LI7000 | P2 prostrate/hemiprostrate dwarf-shrub tundra | 26 ± 9 (2) | [74] |  |
| RU-Che | | Russia | (2003-05): N 68.61304 and E 161.34143  (2013-): N 68,61689 and E 161.35089 | 2003-2004  2013-2016  (April-Nov) | 2003-2004 Open path LI-7500  2013-2016  Closed path LGR-FGGA-24EP | W3 sedge, moss, low-shrub wetland | 45 ± 6 (2) | [75, 76] |  |
| RU-Cok | | Russia | 70.82973 N,  147.48897E | 2003-2013  (n/a) | Open path  LI7500 | W2 sedge moss/dwarf-shrub wetland | n/a | [16] |  |
| RU-Sam | | Russia | 72.3733 N  126.4978E | 2008-2010  2013-2017  (2009-2010  2013-2017) | 2008-2010 Open path LI-7500  2010 and 2013-2017  Closed path LI-7000  2013-2017  Open path LI-7500A | W2 sedge moss/dwarf-shrub wetland | 30 ± 3 (1) | [77, 78, 79] |  |
| CA-DL1 | | Canada | 64.8688553N  111.5747927W | 2004-2019 | 2005-2013 Open path LI7500  2014-2015 Enclosed path LI7200 | S1 erect dwarf-shrub tundra | 35 ± 3 (1) | [15, 80] |  |
| CA-TVC | | Canada | 68.74617N  133.50171W | 2013-2019 | Open path EC-150 | S1 erect dwarf-shrub tundra | 42 ± 2 (1) | [81] |  |

**The average soil moisture indicated in this table includes all the sensors available at the sites; the number of sensors and the soil depths in each of the sites are listed for each site (CA-DL1: N=2 in a wet location and a dry location (both at -10 cm depth); US-Atq: N=4 (2010-2013, at -5 (2),-15, and -30 cm depth), N=12 (2014-2019 at -5 (5),-15 (4), and -30 cm (3) depth; US-Ivo: N= 12 (4 at -5 cm depth, 4 at -15 cm depth, and 4 and -30 cm depth); US-Bes: N= 5 (2 diagonally inserted at 0-10cm, 1 diagonally inserted at -20-30 cm, 2 vertically inserted at 0-30cm depth); US-Che: N=2 (-8cm and -16cm depth); RU-Sam: N=11, 4 in slopes (at -5, -14, -23, -33 depth, and 7 in rims at -5, -12, -15, -22, -26,- 34, and -37 cm depth); US-ICt: N=2 (at -2.5 cm depth); DK-ZaH: N=2 (2000-2004 vertical 0-6 cm and from 2005 onward are at two depths horizontal: -5cm, -10 cm depth) CA-TVC: included one sensor inserted horizontally at -20cm depth. For the rest of the analysis in the paper we used the depth indicated in the methods section in the main manuscript.

**Table S2** Significance (P) and Pearson’s correlation coefficient (r) of the relationships between the indicated monthly median standardized anomalies for June, July, and August retaining site as unit of variation using a partial correlation analysis which regressed the anomalies of the indicated variables while accounting for the anomalies of solar radiation and air temperature, as shown in Fig. S1. Site was retained as the unit of variation by estimating the standardized anomalies by week, month, and site, and then estimating a monthly median of these weekly anomalies for each of the indicated variables, and site. The r was only included when the P<0.1 (given that for P>0.1 we assumed that r is not significantly different from zero), N=284.

| **Regression model** | **month** | **P** | **r** |
| --- | --- | --- | --- |
|  | June | 0.011 | -0.27 |
| NEE ~ soil moist \| Rg & air T | July | 0.0013 | -0.32 |
|  | August | 0.011 | -0.25 |
|  | June | 0.0016 | 0.33 |
| GPP ~ soil moist \| Rg & air T | July | 0.47 | - |
|  | August | 0.99 | - |
|  | June | 0.047 | 0.21 |
| ER ~ soil moist \| Rg & air T | July | 0.51 | - |
|  | August | 0.46 | - |
|  | June | 0.002 | -0.32 |
| soil moist ~ snow melt \| Rg & air T | July | 0.41 | - |
|  | August | 0.21 | - |
|  | June | 0.1 | -0.16 |
| ET ~ snow melt \| Rg & air T | July | 0.37 | - |
|  | August | 0.33 | - |
|  | June | 0.20 | - |
| Bowen ratio ~ snow melt \| Rg & air T | July | 0.62 | - |
|  | August | 0.86 | - |

**Table S3** Mixed effect model between the indicated monthly median standardized anomalies for the indicated variables in June, July, and August including site as random effect, N=284. The model R^2^_m_ was only included when the p-value<0.1.

| **Mixed effect model** | **month** | **p-value** | **R^2^_m_** |
| --- | --- | --- | --- |
|  | June | - | - |
| NEE ~ Rg | July | <0.001 | 0.19 |
|  | August | <0.001 | 0.14 |
|  | June | <0.001 | 0.12 |
| NEE ~ air T | July | - | - |
|  | August | - | - |
|  | June | 0.0077 | 0.078 |
| NEE ~ H | July | <0.001 | 0.24 |
|  | August | 0.078 | 0.032 |
|  | June | - | - |
| NEE ~ Bowen ratio | July | 0.031 | 0.049 |
|  | August | - | - |
|  | June | 0.0138 | 0.065 |
| NEE ~ soil moisture | July | 0.025 | 0.094 |
|  | August | 0.013 | 0.062 |
|  | June | 0.085 | 0.033 |
| NEE ~ LE | July | 0.0010 | 0.11 |
|  | August | <0.001 | 0.14 |
|  | June | - | - |
| NEE ~ VPD | July | - | - |
|  | August | - | - |
|  | June | 0.001 | 0.16 |
| NEE ~ snow melt date | July | 0.015 | 0.062 |
|  | August | <0.001 | 0.17 |

| **Mixed effect model** | **month** | **p-value** | **R^2^_m_** |
| --- | --- | --- | --- |
|  | June | - | - |
| GPP ~ Rg | July | 0.0017 | 0.10 |
|  | August | 0.019 | 0.056 |
|  | June | <0.001 | 0.22 |
| GPP ~ air T | July | 0.0094 | 0.070 |
|  | August | 0.0021 | 0.094 |
|  | June | 0.0015 | 0.11 |
| GPP ~ H | July | <0.001 | 0.11 |
|  | August | - | - |
|  | June | - | - |
| GPP ~ Bowen ratio | July | - | - |
|  | August | - | - |
|  | June | 0.0021 | 0.10 |
| GPP ~ soil moisture | July | - | - |
|  | August | - | - |
|  | June | <0.001 | 0.13 |
| GPP ~ LE | July | 0.032 | 0.050 |
|  | August | - | - |
|  | June | - | - |
| GPP ~ VPD | July | - | - |
|  | August | - | - |
|  | June | <0.001 | 0.24 |
| GPP ~ snow melt date | July | <0.001 | 0.13 |
|  | August | 0.016 | 0.059 |

| **Mixed effect model** | **month** | **p-value** | **R^2^_m_** |
| --- | --- | --- | --- |
|  | June | - | - |
| ER ~ Rg | July | - | - |
|  | August | - | - |
|  | June | <0.001 | 0.20 |
| ER ~ air T | July | 0.001 | 0.15 |
|  | August | 0.001 | 0.16 |
|  | June | 0.081 | 0.033 |
| ER ~ H | July | - | - |
|  | August | - | - |
|  | June | - | - |
| ER ~ Bowen ratio | July | - | - |
|  | August | - | - |
|  | June | 0.048 | 0.043 |
| ER ~ soil moisture | July | - | - |
|  | August | - | - |
|  | June | 0.025 | 0.055 |
| ER ~ LE | July | 0.0012 | 0.11 |
|  | August | - | - |
|  | June | - | - |
| ER ~ VPD | July | 0.0017 | 0.10 |
|  | August | - | - |
|  | June | <0.001 | 0.14 |
| ER ~ snow melt date | July | 0.0012 | 0.11 |
|  | August | - | - |

**Figures**


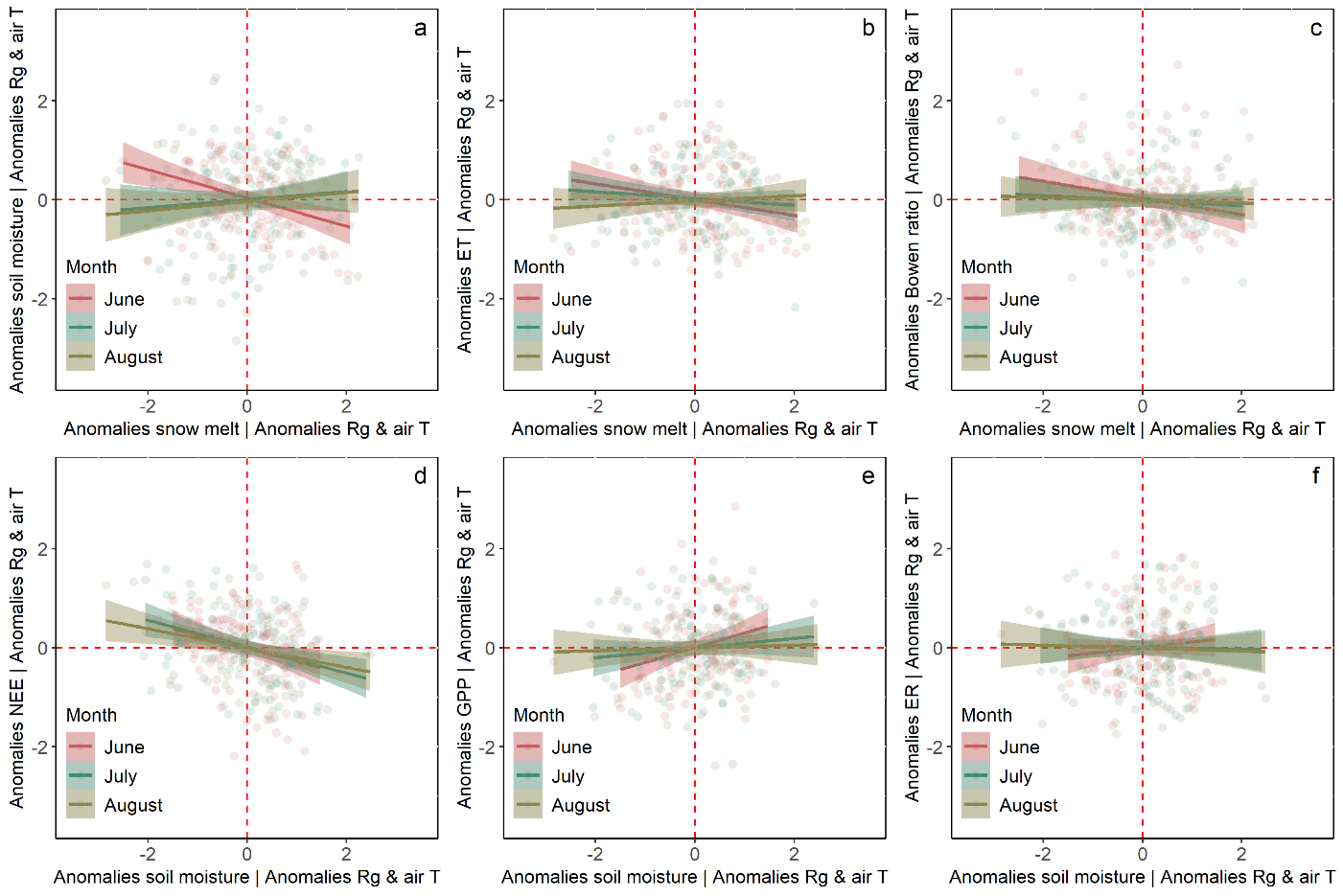


**Fig. S1| Partial correlation between the indicated variables considering the anomalies in solar radiation and air temperature.** Included in the panels the p-value of the indicated partial regressions between the monthly median standardized anomalies indicated in the panels for each of the months, and when significant (P<0.1) included are also the Person’s correlation coefficients in Table S2.


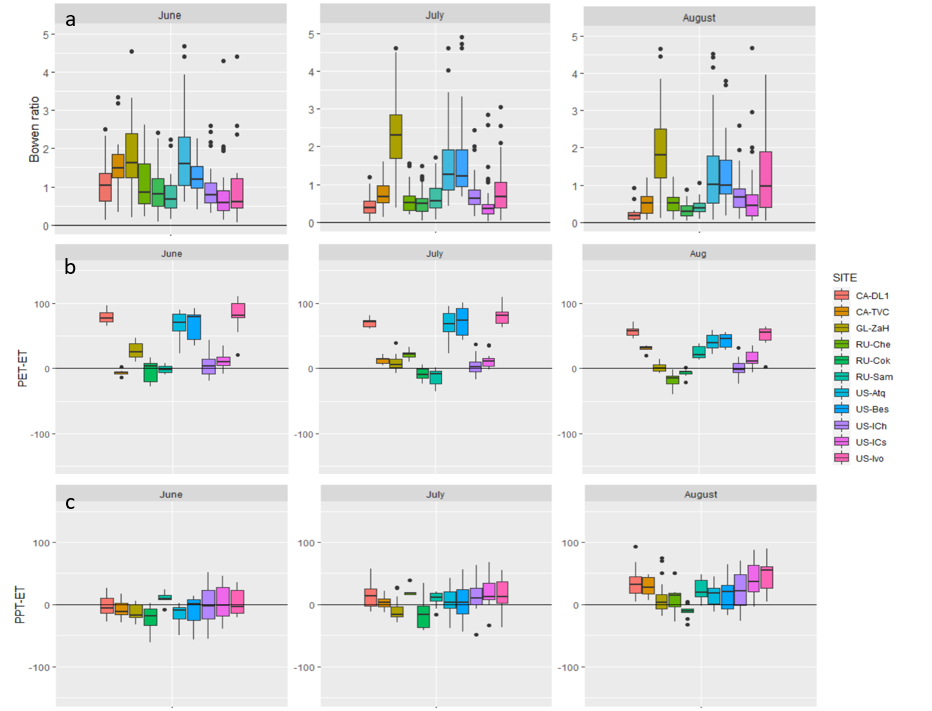


**Fig. S2| Median in the Bowen ratio (a), PPT-ET (mm) (b), and PPT-ET (mm) (c)** for each of the indicated sites and indicated months for the entire period available for each of the sites (Table S1).

**References**

1. Walker, D. A., *et al.* Phytomass, LAI, and NDVI in northern Alaska: Relationships to summer warmth, soil pH, plant functional types, and extrapolation to the circumpolar Arctic. *J. Geophys. Res.-Atmos.* **108**(D2) (2003).
2. Oechel, W. C., Laskowski, C. A., Burba, G., Gioli, B., & Kalhori, A. A. M. Annual patterns and budget of CO_2_ flux in an Arctic tussock tundra ecosystem. J *Geophys. Res.-Biogeo.* 119(3), 323-339, doi:10.1002/2013JG002431 (2014).
3. Zona, D., *et al.* Cold season emissions dominate the Arctic tundra methane budget. *Proc. Natl. Acad. Sci. U.S.A.* **113(1)**, 40-45 (2016).
4. Euskirchen, E. S., Bret-Harte, M. S., Shaver, G. R., Edgar, C. W., & Romanovsky, VE Long-Term Release of Carbon Dioxide from Arctic Tundra Ecosystems in Alaska. *Ecosystems* **20(5)**, 960-974 (2017).
5. Goodrich, J. P., *et al.* Impact of different eddy covariance sensors, site set-up, and maintenance on the annual balance of CO_2_ and CH_4_ in the harsh Arctic environment. *Agr. Forest Meteorol.* **228-229**, 239-251 (2016).
6. Pastorello, G. *et al.* The FLUXNET2015 dataset and the ONEFlux processing pipeline for eddy covariance data. *Sci. Data* **7**, 225, doi:10.1038/s41597-020-0534-3 (2020).
7. Papale, D. & Valentini, R. A new assessment of European forests carbon exchanges by eddy fluxes and artificial neural network spatialization. *Glob. Change Biol.* **9**, 525-535, doi:10.1046/j.1365-2486.2003.00609.x (2003).
8. Hufkens, K. MCD10A1: a robust MODIS snow cover and snow phenology product. <http://doi.org/10.5281/zenodo.162765> (2016).
9. Gascoin, S., *et al.* A snow cover climatology for the Pyrenees from MODIS snow products. *Hydrol. Earth Syst. Sci.* **19(5)**, 2337-2351(2015).
10. Euskirchen, E. S., *et al.* Importance of recent shifts in soil thermal dynamics on growing season length, productivity, and carbon sequestration in terrestrial high-latitude ecosystems. *Glob. Change Biol.* **12(4)**, 731-750 (2006).
11. Kade, A., Bret-Harte, M. S., Euskirchen, E. S., Edgar, C., & Fulweber, R. A. Upscaling of CO_2_ fluxes from heterogeneous tundra plant communities in Arctic Alaska. *J. Geophys. Res.-Biogeo.* **117(G4)** (2012).
12. Lund, M., et al. Trends in CO_2_ exchange in a high Arctic tundra heath, 2000–2010. *J. Geophys. Res.-Biogeo.* **117(G2)**, doi:10.1029/2011jg001901 (2012).
13. Kwon, M. J. *et al.* Drainage enhances modern soil carbon contribution but reduces old soil carbon contribution to ecosystem respiration in tundra ecosystems. *Glob. Change Biol.* 25, 1315-1325, doi:10.1111/gcb.14578 (2019).
14. Göckede, M. *et al.* Negative feedback processes following drainage slow down permafrost degradation. *Glob. Change Biol.* **25**, 3254-3266, doi:10.1111/gcb.14744 (2019).
15. Holl, D., *et al.* A long-term (2002 to 2017) record of closed-path and open-path eddy covariance CO_2_ net ecosystem exchange fluxes from the Siberian Arctic. *Earth Syst. Sci. Data* **11(1)**, 221-240 (2019).
16. Boike, J., *et al.* A 16-year record (2002–2017) of permafrost, active-layer, and meteorological conditions at the Samoylov Island Arctic permafrost research site, Lena River delta, northern Siberia: an opportunity to validate remote-sensing data and land surface, snow, and permafrost models. *Earth Syst. Sci. Data* **11(1)**, 261-299, (2019)
17. Sachs, T., Giebels, M., Boike, J. & Kutzbach, L., Environmental controls on CH4 emission from polygonal tundra on the microsite scale in the Lena river delta, Siberia. *Glob. Change Biol.* **16**, 3096-3110. doi:10.1111/j.1365-2486.2010.02232.x (2010).
18. Lafleur, P. M. & Humphreys, E. R. Spring warming and carbon dioxide exchange over low Arctic tundra in central Canada. *Glob. Change Biol.* **14**, 740–756 (2008).
19. Helbig, M. *et al.* Addressing a systematic bias in carbon dioxide flux measurements with the EC150 and the IRGASON open-path gas analyzers. *Agric. For. Meteorol.* **228-229**, 349-359, doi:https://doi.org/10.1016/j.agrformet.2016.07.018 (2016).
